# Survey of Tick-Borne Zoonotic Agents in *Ixodes* Ticks Carried by Wild Passerines during Postbreeding Migration through Italy

**DOI:** 10.1155/2023/1399089

**Published:** 2023-11-14

**Authors:** Laura Grassi, Giovanni Franzo, Sofia Grillo, Alessandra Mondin, Michele Drigo, Fulvio Barbarino, Cristina Comuzzo, Matteo Legnardi, Michela Bertola, Fabrizio Montarsi, Maria Luisa Menandro

**Affiliations:** ^1^Department of Animal Medicine, Production and Health (MAPS), University of Padua, Viale dell'Università, 16, 35020, Legnaro PD, Italy; ^2^Julian Prealps Nature Park, Piazza del Tiglio, 33010, Resia (UD), Italy; ^3^Istituto Zooprofilattico Sperimentale delle Venezie, Viale dell'Università 10, 35020, Legnaro PD, Italy

## Abstract

Recently, increasing attention has been posed on the role of migrating birds in the spread of ticks and indirectly of tick-borne pathogens (TBPs). Despite, Italy is considered a bridge between continental Europe and North Africa and a necessary path to connect Mediterranean countries, few studies have been conducted on ticks collected from birds migrating through this country and associated TBPs. The aims of this research were to estimate the infestation burden and identify tick species feeding on migratory birds, and perform a molecular screening for TBPs. During autumn migration (2019–2020), birds were inspected for ticks in a ringing station located in north-east Italy. Ticks were identified and screened for tick-borne encephalitis virus (TBEV), *Borrelia burgdorferi* sensu latu, *Rickettsia* spp., *Ehrlichia* spp., *Neoehrlichia* spp., *Anaplasma phagocytophilum*, and *Bartonella* spp. *Ixodes* ticks (*n* = 209) were feeding on 2.6% of passerines (88/3411). Most of these (208/209) were *Ixodes ricinus*, except one *Ixodes frontalis*. Eight bird species were infested: common blackbird, redwing, brambling, song thrush, common chaffinch, European robin, water pipit, and coal tit. *Rickettsiales* showed a low prevalence, from 1.4% of *Ehrlichia* spp., 4.3% of *A. phagocytophilum*, up to 7.2% of *Rickettsia* spp.. *Borrelia burgdorferi* s.l. had the highest prevalence, 54.6%, and several zoonotic genotypes were identified: *B. garinii*, *B. valaisiana*, *B. afzeli*, *B. burgdorferi* sensu stricto, and *B. miyamotoi*. All specimens were negative for TBEV and *Bartonella* spp.. Although the tick burden was generally low, most of the vectors (>60%) were positive for at least one pathogen, highlighting the relevance of a continuous monitoring of migrating birds as potential sources of pathogen dispersal.

## 1. Introduction

Migratory birds are important hosts of several tick species and may spread these ectoparasites over long, medium, or short distances in relation to their migratory movements. Through the dissemination of ticks, birds can potentially spread the associated tick-borne pathogens (TBPs) to other susceptible species in the geographic areas where they move [1]. In addition, wild birds can act as reservoir hosts for some TBPs [2, 3] and may disperse infected ticks that have fed on them to new areas, increasing the likelihood of new foci of disease.

In Europe, most research on wild bird infestation described Ixodid ticks, and in particular the *Ixodes* genus, as the most represented [1, 4, 5]. *Ixodes* ticks, and mainly *Ixodes ricinus* (*I. ricinus*), play a key role in biological cycles of zoonotic pathogens such as tick-borne encephalitis virus (TBEV) [6, 7], *Borrelia burgdorferi* sensu latu (*B. burgdorferi* s.l.), *Rickettsia* spp., *Anaplasma phagocytophilum* (*A. phagocytophilum*) [8], and *Bartonella* spp. in Europe [9].

Previous studies have highlighted the risk of the introduction of TBEV through ticks dispersed by migrating birds [7, 10]. The role of passerines as a TBEV reservoir has been suspected, but their actual epidemiological relevance is not fully known [11–13]. The main reservoirs of TBEV are represented by small mammals such as rodents; other mammals as ungulates are indirectly involved in TBEV epidemiology being pivotal in tick cycle and these latter may disperse ticks on local scale [11]. Contrarily, birds can be infested by ticks while feeding on the ground and then they can spread these parasites to new areas, including the crossing of geographical barriers. Despite tick-feeding time on birds was thought to be shorter than the time spent covering long distances while migrating [11], Fares et al. [14] described a TBEV-European subtype positive-tick in northern Tunisia, probably as a consequence of bird migration in autumn. Similar long-distance dispersal events can thus be expected to occur [10, 15]. Within Western and Central European countries, the main risk is the introduction of high-pathogenic TBEV genotypes, such as the far East and Siberian lineages, through bird movements, as already demonstrated in Finland islands [16]. Although not fully understood, it may be speculated that the tick drop-off does not occur randomly, and that *I. ricinus* may be able to “choose” when to drop-off in a better environmental condition [17].

Migratory movements have also been linked to the presence of other zoonotic agents [18]. Currently, the most widely distributed tick-borne disease in the Northern hemisphere is caused by spirochetes of the *B. burgdorferi* s.l. complex. Borreliae are divided in Lyme group (LG) and relapsing fever group (RFG). Inside the LG, 22 different genospecies have been described with a different zoonotic potential. Indeed, not all of these have been associated with human Lyme disease and, to date, 10 have been isolated from humans, i.e., *B. afzelii*, *B. bavariensis*, *B. bissettii*, *B. burgdorferi* sensu stricto (s.s.), *B. garinii*, *B. kurtenbachii*, *B. lusitaniae*, *B. mayonii*, *B. spielmanii*, and *B. valaisiana* [19]. In Europe, the most prevalent are *B. burgdorferi* s.s., *B. garinii*, and *B. afzelii*, whose reservoir are micromammals or passerine birds. Currently, several genospecies were identified in the ticks feeding on different passerine species that have been suggested both as reservoir and as mechanical carriers [5, 17]. Thrushes (genus *Turdus*) seem to play a role as reservoir for *B. garinii* [2, 20]. The systemic reactivation of *B. garinii* has been demonstrated in association to the autumn migratory restlessness of redwing *Turdus iliacus* (*T. iliacus*) [21]. In Europe, *B. miyamotoi*, an etiological agent of the RFG, was thought to be transmitted mainly by soft ticks. However, it may also be spread and transmitted via hard ticks, especially *I. ricinus* and in the last decades an increasing interest has been posed on this zoonotic bacteria [22]. To date, both bank vole *Myodes glareolus* and yellow-necked mouse *Apodemus flavicollis* are considered competent reservoirs for this bacterium [23]. On the contrary, birds are not considered competent reservoirs, but positive *I. ricinus* ticks feeding on birds have been reported [7, 24].

Spotted fever group (SFG) rickettsiae include several genera that are transmitted through tick-bites; SFG comprises different species and most of them have also been detected in the wild animals, although their sylvatic cycle remains unclear [25]. Indeed, it has been shown that several *Ixodes* species may act both as vectors and reservoirs, displaying an efficient transovarian transmission [26]. The detection of positive samples of blood or tissues of synanthropic (domestic pigeon *Columba livia domestica*) and wild birds (European robin *Erithacus rubecula*), raises questions of birds as potential reservoirs but, to date, there are no certain data for a reliable explanation [27]. On the other hand, migrating birds have been recorded to host different SFG *Rickettsia*-positive ticks, thus being involved as carriers of the positive ectoparasites [1, 28].

*Neoehrlichia mikurensis* (*N. mikurensis*) is an emerging TBP, identified as a human pathogen in 2010 [29]. Although positive (feeding) *I. ricinus* were reported in the several vertebrate species, only micromammals are considered competent reservoir [23, 30]. Indeed, a low prevalence was observed in ticks feeding on birds in the several European countries [31–33].

*Anaplasma phagocytophilum* is an emerging tick-borne pathogen that may cause disease in the different vertebrate species, including humans, dogs, cats, horses and domestic ruminants [34]. In Europe, *A. phagocytophilum* is genetically characterized into four different ecotypes, that display different host ranges. Indeed, Ecotype I infects a broad species range, from birds to mammals, humans included. On the other hand, Ecotypes II, III, and IV have been frequently found in roe deer, small rodents, and wild birds, respectively [35]. Hornok et al. [36] described a bacteriemic *Turdus iliacus* without identifying the ecotype; the role of wild birds in *A. phagocytophilum* infectious cycle remains unclear. To date, *A. phagocytophilum*-positive ticks feeding on birds were found in different European countries as Switzerland, Latvia, Netherlands, and Belgium [7, 31, 37].

*Bartonella* spp. include zoonotic species that are mainly linked to mammal species and flea transmission. A recent study described *Bartonella*-like positivity in birds and associated ectoparasites, widening its susceptible hosts not only within the mammalian group [38]. This finding, together with the evidence of the experimental infection of *B. henselae* in *I. ricinus* ticks, raised questions on ticks as a potential competent vector [39].

In Italy, an important bird migratory route runs through the Eastern Prealps (Friuli Venezia Giulia) bordering Austria and Slovenia [40]. Due to the orographic reasons many migrating birds choose this entering point while moving southwards from north and especially eastern Europe, avoiding the hard crossing of alpine mountains [41]. Therefore, a considerable number of migrating species may be found in Eastern Prealps during autumn, together with resident bird populations that inhabit the area. Given this complex context, and the role of birds in the dissemination of ticks and related pathogens, we conducted a study collecting ticks from birds captured in a ringing station located in this area. The study aimed to improve the knowledge on the occurrence and spread of TBPs by (i) quantifying the bird infestation burden, (ii) identifying the tick species involved, and then (iii) testing all the specimens for TBEV, *B. burgdorferi* s.l., *Rickettsia* spp., *Ehrlichia* spp., *Neoehrlichia* spp., *A. phagocytophilum*, and *Bartonella* spp.

## 2. Materials and Methods

### 2.1. Area of Sampling

The sampling site was located at the “Malga Confin” ringing station (longitude: 46.342, latitude: 13.2179, altitude: 1332m a.s.l.) in the Venzonassa Valley (north-eastern Italy). The station represents an important stopover area, located inside the Julian Prealps Nature Park, and is involved in the Alpi Project (https://progetto-alpi.muse.it/it/), a national project that investigates the postbreeding bird migration during late summer and early autumn.

### 2.2. Samples Collection

For two autumns, 2019 and 2020, all ringed birds were carefully inspected for ectoparasites and data about bird species and the number of animals ringed have been recorded. In addition, for the infested ones, additional data were registered, in detail, age (<1 year, juvenile/>1 year, adult), sex (unsexed/male/female), fat deposition, and muscle development. The total count of ticks found on each animal was registered.

Ticks were removed with tick tweezers and then stored in 70% ethanol either individually or in pools when many specimens were found on the same animal. Tick identification was performed with stereomicroscope and microscope, following Manilla [42] identification and dichotomic keys. To confirm the morphological identification a subset of ticks, including both larvae and nymphs, was analyzed by biomolecular methods.

### 2.3. Biomolecular Analysis

DNA and RNA were coextracted from single ticks using the All Prep DNA/RNA Mini Kit (QIAGEN GmbH, Germany) following the manufacturer's instructions. Before extraction, an internal control for both DNA and RNA provided by Quantinova Pathogen + IC kit (QIAGEN GmbH, Germany) was added according to the manufacturer's instructions. DNA and RNA were stored at −20 and −80°C, respectively. Morphological identification of ticks was confirmed by sequencing a portion of the 16S rRNA gene amplified using the primers described by d'Oliveira et al. [43] on a subset of selected samples. Molecular screenings were performed for the following pathogens: TBEV, *B. burgdorferi* s.l., *Rickettsia* spp., *Ehrlichia* spp., *Neoehrlichia* spp., *A. phagocytophilum*, and *Bartonella* spp. Except for *Neoehrlichia* spp., all pathogens and the internal control, were screened using the Quantinova Pathogen + IC kit (QIAGEN GmbH, Germany) on a LightCycler96 instrument (Roche, Switzerland) with genus-specific real-time PCR assays and the Internal Control Assay provided by the kit manufacturer, respectively. *Neoehrlichia* spp. was screened with an HRM real-time assay using 5x HOT FIREPol EvaGreen qPCR Mix Plus (Solis Biodyne) on MyGo Pro instrument (IT-IS, United Kingdom).

All samples that produced a positive signal in both Internal Control Assay and pathogen screening were further analyzed by specific end-point PCR assays set up using the kit Phire Hot Start II PCRMaster Mix (Thermo Fischer Scientific, USA) and the Biometra TGradient thermal cycler (Analytic Jena GmbH, Germany). The primer and probe sequences, the annealing temperature and the extension time used in the real-time PCR, HRM and end-point PCR assays are summarized in *Supplementary 1*. Positive and negative controls were included in each run.

Amplification products of pathogens and ticks were visualized by electrophoresis on 2% agarose gels stained with SybrSafe DNA Stain (Invitrogen by Thermo Fischer Scientific, USA), and then purified using ExoSap-IT Express PCR Product Cleanup (Thermo Fischer Scientific, USA). Purified amplicons were sent to StarSEQ® GmbH facilities (Mainz, Germany) for bidirectional sequencing using the same primers used in the PCR assay. Consensus nucleotide sequences, assembled and edited using ChromasPro v.2.1.8 (Technelysium Pty Ltd., Australia), were deposited in Genbank (accession number details, *Supplementary 2*) and analyzed using the Nucleotide BLAST search engine (National Center for Biotechnology Information, Maryland, United States) [44].

### 2.4. Phylogenetic Analysis

The obtained *Borrelia miyamotoi* sequences were submitted to BLAST analysis and a collection of closely related sequences was identified and downloaded from Genbank [45]. Collection country and host were annotated in the sequence name when available. Selected sequences were aligned with the ones obtained in the present study using MAFFT [46]. A neighbor joining tree was reconstructed using MEGA X [47] selecting as substitution model the one with the lowest Akaike information criterion (AIC), calculated with JModeltest [48]. The reliability of inferred clades was inferred by performing 1,000 bootstrap replicates. The ecotype of *A. phagocytophilum* sequences was identified by aligning them with the reference dataset available in Grassi et al. [49] and performing phylogenetic analysis as previously described.

## 3. Results

### 3.1. Migratory Birds

A total of 3411 birds belonging to 46 species were ringed in the “Malga Confin” ringing station. Most of the animals were captured in 2019 (2734 birds of 42 bird species) compared to 2020 (677 birds of 32 bird species), due to the harsh weather conditions occurred during the latter year. In 2019, the weather conditions allowed working 36 out of 40 days. Most of the trapped species were recorded in both years, with some exceptions. In both years of sampling, 17.4% (8/46) of captured species and 2.6% (88/3,411) of total birds crossing Italian borders were found to be infested with ticks. Details of the sampled species and the precise number of captures are provided in *Supplementary 3*. Common blackbird (*Turdus merula*) showed the highest infestation rate 42.5% (37/87), followed by redwing (*T. iliacus*) 26.7% (4/15) and brambling (*Fringilla montifringilla*) 14,0% (13/93); all the remaining species, i.e., song thrush (*Turdus philomelos*), common chaffinch (*Fringilla coelebs*), European robin (*Erithacus rubecula*), water pipit (*Anthus spinoletta*), and coal tit (*Periparus ater*) showed lower infestation rates. Detailed results are provided in Table 1 and in *Supplementary 3*.

Ringed and infested birds were mainly first year individuals (60/88); sex identification occurred as follows: males 36/88, females 19/88, and not recognizable 33/88.

Overall, animals in which at least one infected tick was detected had a tendency toward a lower fat deposition (mean = 1.13; standard error = ± 0.12) and muscle (mean = 1.81; standard error = ± 0.06) development than birds carrying pathogen-negative ticks (i.e., fat score = 1.77 ± 0.15 and muscle score = 1.95 ± 0.03). The number of ticks or engorgement status of ticks did not seem related to these parameters, although the features of the study prevented a formal assessment. The total count of ticks found on each animal was also registered. A maximum of 11 ticks were found on a single bird (common blackbird). Most of the cofeeding ticks were found on three bird species, common blackbird, brambling, and European robin. Details on the number of ticks collected from each species are reported in Table 2.

### 3.2. Ticks

Overall, 209 ticks were collected and identified as immature stages, i.e., larvae (*n* = 48) and nymphs (*n* = 161). The most prevalent species was *Ixodes ricinus* (*n* = 208, 99.5%). One *Ixodes frontalis* larva (confirmed by molecular analysis) was collected from a European robin.

All the nucleic acids extracted from ticks were positive for both DNA and RNA internal control assays, highlighting the extraction efficiency and the absence of PCR inhibitors.

Different host species had a different infestation frequency, where the common blackbird and redwing were more commonly infested compared to the other species like robin (Table 1). No clear pattern was found based on the animal age or sex. *Ixodes frontalis* tested negative to all pathogens and thus the following results refer all to *I. ricinus*.

All *I. ricinus* positive to *Rickettsia* (15/209%–7.2% (CI: 4.4%–11.5%)) were nymphs and harbored *Rickettsia helvetica (R. helvetica)*. Ticks were collected from 5 out of 8 considered bird species (common blackbird, redwing, European robin, common chaffinch, and brambling).

*Anaplasma phagocytophilum* was detected in 9 out of 209 ticks, i.e., eight nymphs and one larva of *I. ricinus* (4.3% (CI: 2.0%–8.0%)). Almost all of them were collected from common blackbirds (*n* = 7) while the others were found on brambling (*n* = 1) and water pipit (*n* = 1). Good quality sequences, obtained from the assembly of forward and reverse reads, were obtained from four samples, and further analysis demonstrated that these strains belong to the Ecotypes I and II (*Supplementary 4*).

*Ehrlichia* spp. was identified in 3 out of 209 ticks, which were all *I. ricinus* nymphs (1.4% (CI: 0.3%–4.1%)). Positive ticks were collected from two common blackbirds and one European robin. In detail, they were identified as *Ehrlichia muris* (1/3) and *N. mikurensis* (2/3). Most of the ticks positive to *Rickettsia*, *Anaplasma*, *Ehrlichia*, and *Neoehrlichia* have been collected as single ticks infesting one single bird.

*Borrelia burgdorferi* s.l. was identified in 114 out of 209 ticks at screening (54.6% (CI: 47.5%–61.4%)), mostly in nymphs (100/161) 62.1% (CI: 54.1%–69.6%) compared to larvae (14/48) 29.2% (CI: 17.0%–44.0%).

**Figure 1:**
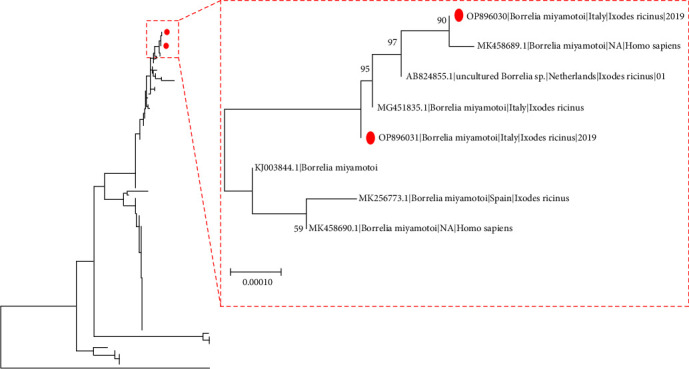
Evolutionary tree of *Borrelia miyamotoi* strains inferred using the neighbor-joining method on a dataset of partial *groEL* sequences. The confidence probability was estimated using the bootstrap test. Only values higher than 70% are reported. The strains identified in the present study are magnified in the right insert and highlighted by red circles. A complete figure of the tree is available in *Supplementary 5*.

Most of them were identified as *B. garinii* (*n* = 64) and derived from *I. ricinus* nymphs (*n* = 56) and larvae (*n* = 8) collected as single specimens or while cofeeding in common blackbirds, brambling, song thrush, and redwing. *Borrelia valaisiana* (*n* = 23) was detected in both immature stages of *I. ricinus* (21 nymphs and 2 larvae), while feeding or cofeeding on common blackbird, brambling, and song thrush. *Borrelia afzelii* (*n* = 5) was only identified in *I. ricinus* nymphs feeding on common blackbird and common chaffinch, while *B. burgdorferi* s.s. (*n* = 2) was identified in a larva and a *I. ricinus* nymph feeding on common blackbirds. Of note, two specimens were positive to RFG Borrelia, i.e., *B. miyamotoi* (*n* = 2). Both ticks were *I. ricinus* nymphs feeding on a common chaffinch and an European robin and the identified strains clustered together with Europe-derived sequences of both human and animal origin (Figure 1 and *Supplementary 5*).

TBEV and *Bartonella* spp. were not detected in any sample.

Overall, 15 out of 209 (7.2% (CI: 4.1%–11.6%)) ticks were found to be coinfected by two pathogens. Most of the associations involved *R. helvetica* and different genotypes of *B. burgdorferi* s.l. but, in addition, several LG spirochetes were also found in association with *A. phagocytophilum* and *N. mikurensis*. Coinfected specimens were collected from 1 brambling, 1 European robin, and 12 common blackbirds; of note, two nymphs cofeeding on the same common blackbird, were both coinfected with *R. helvetica* and *B. garinii*.

## 4. Discussion

Different bird species, captured in the ringing station in the Eastern Prealps, were hosting *Ixodes* ticks. Compared to the other European studies, our data show a low-tick infestation rate in birds, around 2.6%, compared to the higher values found in Switzerland (11.4%), Denmark (10.7%), and Slovakia (26.5%) [1, 7, 32]. On the other hand, despite the lower number of ticks, more than half of the specimens (approx. 60%) were positive for at least one TBP. Similar to the previous studies, most of the birds were infested by *Ixodes* spp. Out of the 209 ticks, only one tick was identified as *I. frontalis*, an ornithophilic species associated with the presence of passerines [50, 51]. The other tick species was *I. ricinus* and we confirm that birds are predominantly hosts for immature (larvae and nymphs) *I. ricinus* stages, in particular thrushes that display a ground feeding behavior [1, 5, 52]. Moreover, *I. ricinus* ticks were associated to *B. burgdorferi* s.l. positivity that counted the highest prevalence in this study, confirming the association between *I. ricinus*, passerine birds and *Borrelia* positivity in Europe [53]. In North America, an association between the geographic range of Lyme diseases and the bird migration flyways was previously suspected [18]. The present study reports a higher prevalence (54.6%) of *Borrelia*-positive ticks, compared with the studies included in a recent review [5] but the presence of a high percentage of infested thrushes — common blackbirds, redwing, and song thrush — in the considered ringing station could have played a role in the total prevalence. As reported by Rataud et al. [54] *Borrelia* prevalence in feeding ticks represents a good proxy for the avian realized reservoir competence (i.e., *Borrelia* prevalence in birds, bird infectivity, and systemic/nonsystemic transmission). The most reliable indirect way to assess these aspects is to consider the positivity of feeding larvae. Assuming that *Borrelia* spp. transovarial transmission usually occurs at a low rate [26], the considerable number of infected feeding larvae underlies a probable reservoir competence [54]. According to our results, 14 out of the 48 larvae were positive and collected mostly from common blackbirds (9), and few other individuals (one song thrush, one redwing, and one brambling); 34 negative larvae were collected from different species (14 robins, 4 common blackbirds, 4 bramblings, and 1 common chaffinch). Common blackbird and redwing species had the highest frequency of tick infection; this finding is in line with a previous research that evidenced the involvement of these two species as competent reservoirs [20] according to this, no link was described with other migrating species. The genospecies *B. garinii* (*n* = 64) and *B. valaisiana* (*n* = 23) accounted for most of the identifications, confirming the link between these species and wild passerines [5, 19, 55, 56]. In addition, finding *B. afzelii* in feeding nymphs may be related to a previous meal on small rodents, which are considered competent reservoirs of this genospecies [5, 56, 57]. Thus, the detection of positive *B. afzelii* ticks feeding on birds suggests them as mechanical carriers. Conversely, other authors found positive *B. afzelii* tissues in common blackbirds but, despite this evidence, their concrete role has not been assessed yet [19, 57]. In the present study, we have not screened passerine blood knowing that it is not a sensitive matrix and that *Borreliae* are rarely detected in blood [58]. However, seeing the overall high-*Borrelia* prevalence in feeding ticks from thrushes a systemic infection cannot be ruled out.

Of note, an apparent effect of tick infestation on fat and muscle score was observed, which suggests a relation between a worse nutritional status and *Borrelia*-positive ticks feeding on them. However, the limited sample size and the presence of confounding factors prevent robust conclusions. Ethological factors could affect the association between infection and fat/muscle score, as thrushes with low-fat score may search for food on bushes, trees, and also in the ground to regain fat and weight. Although thrushes are not exclusively ground-feeders, the possibility that tick infestation could be increased by ground-feeding cannot be ruled out; in addition, high stress induced by migration may also play a role in immune suppression and a reduced capacity to cope with infective agents [21]. For this reason, further dedicated studies should be performed to improve the knowledge about this issue. Related to RFG positivity, a different scenario can be drawn for *B. miyamotoi*, which is considered a rodent-associated genospecies, although efficient transovarial transmission has also been proven [59]. To date, this species has been rarely found in bird tissues and the competence as reservoir has been experimentally confirmed only in micromammals [59]. Indeed, positive *B. miyamotoi* ticks found on birds are a probable incidental finding [7, 24]. The two positive specimens of the present study were genetically related to European type sequences (*Supplementary 5* and Figure 1). In detail, one of the two sequences had the highest identity with a strain reported in an infected patient from Sweden, while the other were highly similar to a sequence found in a *I. ricinus* tick in northern Italy. Like *B. miyamotoi* epidemiology, *I. ricinus* nymphs positive for *E. muris* and *N. mikurensis* are a probable result of a previous meal on micromammals, considered reservoir of both these species [23, 60]. Heylen et al. [31] found a higher prevalence of *N. mikurensis* (4%) compared to our results but as reported in the other studies, the prevalence in ticks feeding on birds is generally low [31–33]. This is related to the role of avian species that, to date, are not considered competent reservoirs. A 4.3% overall prevalence of *A. phagocytophilum* was found in screened ticks. Previous studies reported a prevalence from 0.2% up to 13.2% in ticks from birds in different European countries [7, 31, 37]. In our study, no significant association between *A. phagocytophilum* positivity and bird host species was highlighted. Phylogenetic analysis did not reveal any Ecotype IV for which avian species are thought to be reservoir [35]. Indeed, two *A. phagocytophilum* sequences from *I. ricinus* nymphs found on common blackbirds were identified as Ecotype I, that has a broad host range, comprising several mammals and humans, while another two were collected from a common blackbird and a brambling and classified as Ecotype II (further details in *Supplementary 4*). Phylogenetic analysis revealed a high similarity of three out of the four *A. phagocytophilum* strains with other obtained from *I. ricinus* ticks and ungulates' blood collected from neighboring areas of the “Malga Confin” ringing station [49]; the infestation of migrating birds while recovering during the stopover could have occurred. On the other hand, the remaining *A. phagocytophilum* Ecotype I strain had a high homology with a sequence deriving from a cattle blood sample collected in Germany (GQ452230, unpublished data), suggesting a potential relation between strains. Previous studies conducted in Czech Republic also found Ecotype I, in bird tissues, supporting that avian hosts may become infected [61]. Our results revealed the presence of *R. helvetica* in *I. ricinus* feeding on birds (7.2%). A similar prevalence (10%) was reported in a previous Italian study, that identified *R. helvetica* and other SFG rickettsiae [62]. Other studies found a similar prevalence in Denmark and Switzerland, with a high rate of *R. helvetica* identification among all the other genera [7, 32]. Hornk et al. [27, 36] found a higher prevalence of rickettsiae in bird-feeding ticks (51,4%); in addition, despite their description of *R. helvetica* in a blood sample collected from a robin, to date the actual role of wild species in rickettsiae sylvatic cycle has not been clarified. Accordingly, the conducted statistical analysis did not reveal any significant association with bird species and SFG rickettsiae. *Bartonella* spp. was not detected in any sample. A previous study reported the same results [63]. Although *I. ricinus* ticks were suspected as *Bartonella* vectors, their actual role seems negligible, at least in ticks feeding on birds [39]. Indeed, the only evidence of the association between *Bartonella* and migratory birds derives from a previous study, that described positivity in three migratory species and their related nest ectoparasites (*Dermanyssus*, *Ceratophyllus*, and *Protocalliphora*) but not in the ticks [38]. No positivity for TBEV was recorded in the present study. However, other studies found a variable TBEV prevalence in ticks from 0% up to 14% [13, 15, 52, 64]. Moreover, in Italy most of the TBEV-EU subtypes share a common origin, apart from a recent isolation genetically related to Central Europe subtypes [10]. Thus, the risk of new TBEV-subtypes introduction and the monitoring of migrating birds should be taken into account. Coinfections were also found, up to 7.2%, confirming the likelihood of dispersal through birds of ticks positive for several pathogens, creating further threat for human and animal health.

## 5. Conclusions

The present study detected several TBPs, comprising *B. burgdorferi* sl. complex, *B. miyamotoi*, *A. phagocytophilum*, *Rickettsia* spp., and *Ehrlichia* spp., in ticks feeding on several species of migratory birds captured in a ringing station located in north-east Italy. The detection of these pathogens, and the phylogenetic similarities with human-derived samples, confirmed how these multihost pathogens are interconnected between different host species and countries, representing a threat for wild and domestic animals, and human beings as well. Although it is not possible to infer the precise origin of sampled avian species, a probable mixture of both resident and migratory subpopulations may have been sampled in the ringing station during the autumn migration. Therefore, this research pointed out the importance of monitoring wild populations and the role of ringing stations in obtaining epidemiological data useful in surveillance programs.

## Figures and Tables

**Table 1:** Descriptive data about infested migratory birds.

Bird species^a^	Infested/ringed	Infested/ringed	Infested/ringed	
	2019	2020	Total	Infestation rate (%)
Water pipit (*Anthus spinoletta*)	1/42	0/7	1/49	2.0 (CI: 0.1–10.9)
European robin (*Erithacus rubecula*)	11/776	12/197	23/973	2.4 (CI: 1.5–3.5)
Blackbird (*Turdus merula*)	29/67	8/20	37/87	42.5 (CI: 32.0–53.6)
Song thrush (*Turdus philomelos*)	3/65	1/12	4/77	5.2 (CI: 1.4–12.8)
Redwing (*Turdus iliacus*)	3/14	1/1	4/15	26.4 (CI: 7.8–55.1)
Coal tit (*Periparus ater*)	0/884	1/13	1/897	0.1 (CI: 0–0.6)
Common chaffinch (*Fringilla coelebs*)	3/104	2/37	5/141	3.6 (CI: 1.2–8.1)
Brambling (*Fringilla montifringilla*)	13/90	0/3	13/93	14.0 (CI: 7.7–22.7)
Total	63/2042	25/290	88/2332	2.6^b^

*Notes*: ^a^Data about ringed but NOT infested species are reported in the *Supplementary 3*. ^b^Infestation rate counting total captured birds and not only infested species. The partial (2019 and 2020) and total count of ringed and infested animals are reported, together with the infestation rate of each bird species and total.

**Table 2:** Table reporting the number of ticks found per single bird, depending on the considered bird species.

	Number of ticks per bird									
Infested birds	1	2	3	4	5	6	7	10	11	Total
Coal tit (*Periparus ater*)	1									1
Common chaffinch (*Fringilla coelebs*)	5									5
Common blackbird (*Turdus merula*)	11	7	4	4	4	1	4	1	1	37
Brambling (*Fringilla montifringilla*)	9		1		2		1			13
European robin (*Erithacus rubecula*)	16	5	1	1						23
Water pipit (*Anthus spinoletta*)	1									1
Song thrush (*Turdus philomelos*)	4									4
Redwing (*Turdus iliacus*)	2		2							4
Total	49	12	8	5	6	1	5	1	1	88

## Data Availability

All data generated and analyzed during this study are included in this research article and its supporting information files. Further information may be available on request from the corresponding author.

## Supplementary Materials

Supplementary 1 Information about the detection tests used for each pathogen. Details about bacterial/viral species, method used, target genes, primers, probes, and bibliography references.

Supplementary 2 Details about deposited sequences in Genbank: accession number, target gene, tick species, bird host, and bacterial species identified.

Supplementary 3 Ringed bird species during season 2019, 2020, and total count. Details are provided about the partial and total number of bird species and the quantity of individuals/species. Tick infested bird species were orange-labelled.

Supplementary 4
*Anaplasma phagocytophilum* phylogenetic tree. The evolutionary tree was inferred using the neighbor-joining method on a dataset of partial *groEL* sequences. The confidence probability was estimated using the bootstrap test. Only values higher than 70% are reported. The strains identified in the present study are highlighted by red circles.

Supplementary 5
*Borrelia miyamotoi* phylogenetic tree. The evolutionary tree was inferred using the neighbor-joining method on a dataset of partial *groEL* sequences. The confidence probability was estimated using the bootstrap test. Only values higher than 70% are reported. The strains identified in the present study are magnified in the right insert and highlighted by red circles.
