# Supplementary material for: Survey of Tick-Borne Zoonotic Agents in Ixodes Ticks Carried by Wild Passerines during Postbreeding Migration through Italy
Source: Transbound Emerg Dis. 2023 Nov 14;2023:1399089. doi: 10.1155/2023/1399089 (PMC12016753; doi:10.1155/2023/1399089)
Supplement: Supplementary 1 — Information about the detection tests used for each pathogen. Details about bacterial/viral species, method used, target genes, primers, probes, and bibliography references. [file 1399089.f1.pdf]

Species	Method	Target gene	Primer and probe sequences	Ref	Thermal profile (Temp °C/time s)
TBEV	Real-time PCR	NS'3	TBE-f: TGGGCGGTTCTTGTTCTCC	11	95°C/5 s<br>60°C/30 s
			TBE-r: TCACACATCACCTCCTTGTCAGA		
			TBE-FAM: CTGAGCCACCATCACCCAGACACAG		
B. burgdorferi s.l.	Real-time PCR	23S rRNA	Bb23Sf: CGAGTCTTAAAAGGGCGATTTAGT	2	95°C/5 s<br>60°C/30 s<br>72°C/30 s
			Bb23Sr: GCTTCAGCCTGGCCATAAATAG		
			Bb23Sp-FAM: AGATGTGGTAGACCCGAAGCCGAGTG		
	PCR	*gro*EL	GF: TACGATTTCTTATGTTGAGGG	9	98 °C 5 s<br>59->57/°C<br>(-0,5°C/cycle)/5 s<br>72°C/7 s
			GR: CATTGCTTTTCGTCTATCACC		
B. miyamotoi	PCR	*glp*Q	glpQ-f: ATGGGTTCAAACAAAAAGTCACC	6	98 °C 5 s<br>70->60/°C<br>(-0,5°C/cycle)/5 s<br>72°C/15 s
			glpQ-r: CATTACTGTGTCAGTAAAATCTGTAAATATA CCATCTAC		
Rickettsia spp.	Real-time PCR	*glt*A	RKND03f: GTGAATGAAAGATTACACTATTTAT	10	98°C/5 s<br>60°C/30 s
			RKND03r: GTATCTTAGCAATCATTCTAATAGC		
			RKND03-FAM: CTATTATGCTTGCGGCTGTCGGTTC		
	Nested PCR	*glt*A-outer	RpCS.877p: GGGGGCCTGCTCACGGCGG	1	98°C/5 s<br>54°C/5 s<br>72°C/7 s
			RpCS.1258n: ATTGCAAAAAGTACAGTGAACA		
		*glt*A-inner	RpCS.896p: GGCTAATGAAGCAGTGATAA		98°C/5 s<br>54°C/5 s<br>72°C/7 s
			RpCS.1233n: GCGACGGTATACCCATAGC		
Ehrlichia spp.	PCR	16S rRNA	PER1: TTTATCGCTATTAGATGAGCCTATG	7	95°C/5 s<br>65->58°C<br>(-0,5°C/cycle)/5 s<br>72°C/7 s
			PER2: CTCTACACTAGGAATTCCGCTAT		
Candidatus Neoehrlichia mikurensis	HRM	16S rRNA	F: GGGGATGATGTCAARTCAGCAY	8	95°C/15 s<br>60°C/20 s<br>72°C/20 s
			R: CACCAGCTTCGAGTTAAGCCAAT		
Anaplasma phagocytophilum	Real-time PCR	*msp*2	ApMSP2f: ATGGAAGGTAGTGTTGGTTATGGTATT	2	95°C/5 s<br>60°C/5 s<br>72°C/30 s
			ApMSP2r: TTGGTCTT GAAGCGCTCGTA		
			ApMSP2p-HEX: TGGTGCCAGGGTTGAGCTTGAGATTG		
	PCR	*gro*EL	groEL-643s: ACTGATGGTATGCARTTTGAYCG	5	98°C/5 s<br>56°C/5 s<br>72°C/7 s
			groEL-1236as: TCTTTRCGTTCYTTMACYTCAACTTC		
Bartonella spp.	Real-time PCR	*ssr*A	ssrA-F: GCTATGGTAATAAATGGACAATGAAATAA	3	95°C/5 s<br>60°C/30 s

			ssrA-R: GCTTCTGTTGCCAGGTG<br>ssrA-FAM: ACCCCGCTT AAACCTGCGACG		
Tick species	PCR	16S rRNA	16S+1: CTGCTCAATGATTTTTTAAATTGCTGTGG<br>16S-1: CCGGTCTGAACTCAGATCAAGT	4	98°C/5 s<br>64->54°C<br>(-0,5°C/cycle)/5 s<br>72°C/7s

1 Choi, Y.J., W.J. Jang, J.H. Kim, J.S. Ryu, S.H. Lee, K.H. Park, H.S. Paik, Y.S. Koh, M.S. Choi, and I.S. Kim, 2005: Spotted fever group and typhus group rickettsioses in humans, South Korea. *Emerg. Infect. Dis.* **11**, 237–244, DOI: 10.3201/eid1102.040603.

2 Courtney, J.W., L.M. Kostelnik, N.S. Zeidner, and R.F. Massung, 2004: Multiplex real-time PCR for detection of Anaplasma phagocytophilum and Borrelia burgdorferi. *J. Clin. Microbiol.* **42**, 3164–3168, DOI: 10.1128/JCM.42.7.3164-3168.2004.

3 Diaz, M.H., Y. Bai, L. Malania, J.M. Winchell, and M.Y. Kosoy, 2012: Development of a Novel Genus-Specific Real-Time PCR Assay for Detection and Differentiation of Bartonella Species and Genotypes. *J. Clin. Microbiol.* **50**, 1645–1649, DOI: 10.1128/JCM.06621-11.

4 D'Oliveira, C., van Der Weide, M., Jacquiet, P. & Jongejan, F. (1997). Detection of *Theileria annulata* by the PCR in ticks (Acari: Ixodidae) collected from cattle in Mauritania. *Experimental and Applied Acarology*, *21*(5), 279–291. https://doi.org/10.1023/a:1018455223462

5 Guillemi, E.C., L. Tomassone, and M.D. Farber, 2015: Tick-borne Rickettsiales: Molecular tools for the study of an emergent group of pathogens. *J. Microbiol. Methods* **119**, 87–97, DOI: 10.1016/j.mimet.2015.10.009.

6 Hovius, J.W.R.R., B. De Wever, M. Sohne, M.C. Brouwer, J. Coumou, A. Wagemakers, A. Oei, H. Knol, S. Narasimhan, C.J. Hodiamont, S. Jahfari, S.T. Pals, H.M. Horlings, E. Fikrig, H. Sprong, and M.H.J.J. Van Oers, 2013: A case of meningoencephalitis by the relapsing fever spirochaete Borrelia miyamotoi in Europe. *Lancet* **382**, 658, DOI: 10.1016/S0140-6736(13)61644-X.

7 Inokuma, H., K. Ohno, T. Onishi, D. Raoult, and P. Brouqui, 2001: Detection of Ehrlichial Infection by PCR in Dogs from Yamaguchi Okinawa Prefectures, Japan. *J. Vet. Med. Sci.* **63**, 815–817, DOI: 10.1292/jvms.63.815.

8 Krücken, J., C. Schreiber, D. Maaz, M. Kohn, J. Demeler, S. Beck, E. Schein, P. Olias, D. Richter, F.R. Matuschka, S. Pachnicke, K. Krieger, B. Kohn, and G. Von Samson-Himmelstjerna, 2013: A novel high-resolution melt PCR assay discriminates Anaplasma phagocytophilum and "Candidatus Neoehrlichia mikurensis." *J. Clin. Microbiol.* **51**, 1958–1961, DOI: 10.1128/JCM.00284-13.

9 Lee, S.H., J.H. Lee, H.S. Park, W.J. Jang, S.E. Koh, Y.M. Yang, B.J. Kim, Y.H. Kook, and K.H. Park, 2003: Differentiation of Borrelia burgdorferi sensu lato through groEL gene analysis. *FEMS Microbiol. Lett.* **222**, 51–57, DOI: 10.1016/S0378-1097(03)00237-4.

10 Rolain, J.M., I. Bitam, S. Buffet, J.L. Marié, O. Bourry, C. Portelli-Clerc, J.C. Beaucournu, P. Parola, P.E. Fournier, B. Davoust, and D. Raoult, 2009: Presence or absence of plasmid in Rickettsia felis depending on the source of fleas. *Eur. Soc. Clin. Microbiol. Infect. Dis.* **15 Suppl 2**, 296–297, DOI: 10.1111/j.1469-0691.2008.02245.x.

11 Schwaiger, M., and P. Cassinotti, 2003: Development of a quantitative real-time RT-PCR assay with internal control for the laboratory detection of tick borne encephalitis virus (TBEV) RNA. *J. Clin. Virol.* **27**, 136–145, DOI: 10.1016/s1386-6532(02)00168-3.
