# Supplementary material for: Survey of Tick-Borne Zoonotic Agents in Ixodes Ticks Carried by Wild Passerines during Postbreeding Migration through Italy
Source: Transbound Emerg Dis. 2023 Nov 14;2023:1399089. doi: 10.1155/2023/1399089 (PMC12016753; doi:10.1155/2023/1399089)
Supplement: Supplementary 2 — Details about deposited sequences in Genbank: accession number, target gene, tick species, bird host, and bacterial species identified. [file 1399089.f2.pdf]

Accession number	Gene	Tick Species	Source	Bacterial species	Year
OP895930	*groEL*	*Ixodes ricinus*	Song thrush	*B. garinii*	2019
OP895931	*groEL*	*Ixodes ricinus*	Blackbird	*B. garinii*	2019
OP895932	*groEL*	*Ixodes ricinus*	Blackbird	*B. garinii*	2019
OP895933	*groEL*	*Ixodes ricinus*	Blackbird	*B. garinii*	2019
OP895934	*groEL*	*Ixodes ricinus*	Blackbird	*B. garinii*	2019
OP895935	*groEL*	*Ixodes ricinus*	Blackbird	*B. garinii*	2019
OP895936	*groEL*	*Ixodes ricinus*	Blackbird	*B. garinii*	2019
OP895937	*groEL*	*Ixodes ricinus*	Blackbird	*B. valaisiana*	2019
OP895938	*groEL*	*Ixodes ricinus*	Blackbird	*B. garinii*	2019
OP895939	*groEL*	*Ixodes ricinus*	Blackbird	*B. garinii*	2019
OP895940	*groEL*	*Ixodes ricinus*	Blackbird	*B. garinii*	2019
OP895941	*groEL*	*Ixodes ricinus*	Blackbird	*B. afzelii*	2019
OP895942	*groEL*	*Ixodes ricinus*	Blackbird	*B. valaisiana*	2019
OP895943	*groEL*	*Ixodes ricinus*	Blackbird	*B. valaisiana*	2019
OP895944	*groEL*	*Ixodes ricinus*	Blackbird	*Borrelia sp.*	2019
OP895945	*groEL*	*Ixodes ricinus*	Blackbird	*B. garinii*	2019
OP896030	*glpQ*	*Ixodes ricinus*	Chaffinch	*B. miyamotoi*	2019
OP896032	*groEL*	*Ixodes ricinus*	Chaffinch	*B. miyamotoi*	2019
OP895946	*groEL*	*Ixodes ricinus*	Blackbird	*B. garinii*	2019
OP895947	*groEL*	*Ixodes ricinus*	Blackbird	*B. garinii*	2019
OP895948	*groEL*	*Ixodes ricinus*	Blackbird	*B. garinii*	2019
OP895949	*groEL*	*Ixodes ricinus*	Blackbird	*B. garinii*	2019
OP895950	*groEL*	*Ixodes ricinus*	Blackbird	*B. garinii*	2019
OP895951	*groEL*	*Ixodes ricinus*	Blackbird	*B. garinii*	2019
OP895952	*groEL*	*Ixodes ricinus*	Blackbird	*B. garinii*	2019
OP895953	*groEL*	*Ixodes ricinus*	Blackbird	*B. garinii*	2019
OP895954	*groEL*	*Ixodes ricinus*	Blackbird	*B. garinii*	2019
OP895955	*groEL*	*Ixodes ricinus*	Blackbird	*B. garinii*	2019
OP895956	*groEL*	*Ixodes ricinus*	Blackbird	*B. garinii*	2019
OP895957	*groEL*	*Ixodes ricinus*	Blackbird	*B. valaisiana*	2019

OP895958	*groEL*	*Ixodes ricinus*	Blackbird	*B. burgdorferi*	2019
OP895959	*groEL*	*Ixodes ricinus*	Blackbird	*B. garinii*	2019
OP896031	*glpQ*	*Ixodes ricinus*	European robin	*B. miyamotoi*	2019
OP896033	*groEL*	*Ixodes ricinus*	European robin	*B. miyamotoi*	2019
OP895960	*groEL*	*Ixodes ricinus*	Blackbird	*B. valaisiana*	2019
OP895961	*groEL*	*Ixodes ricinus*	Brambling	*B. garinii*	2019
OP895962	*groEL*	*Ixodes ricinus*	Brambling	*B. garinii*	2019
OP895963	*groEL*	*Ixodes ricinus*	Brambling	*B. valaisiana*	2019
OP895964	*groEL*	*Ixodes ricinus*	Brambling	*B. valaisiana*	2019
OP895965	*groEL*	*Ixodes ricinus*	Brambling	*B. garinii*	2019
OP895966	*groEL*	*Ixodes ricinus*	Blackbird	*B. garinii*	2019
OP895989	*groEL*	*Ixodes ricinus*	Blackbird	*B. garinii*	2019
OP895990	*groEL*	*Ixodes ricinus*	Blackbird	*B. garinii*	2019
OP895967	*groEL*	*Ixodes ricinus*	Blackbird	*B. garinii*	2019
OP895968	*groEL*	*Ixodes ricinus*	Blackbird	*B. valaisiana*	2019
OP895991	*groEL*	*Ixodes ricinus*	Blackbird	*B. valaisiana*	2019
OP895969	*groEL*	*Ixodes ricinus*	Blackbird	*B. garinii*	2019
OP895970	*groEL*	*Ixodes ricinus*	Blackbird	*B. garinii*	2019
OP895971	*groEL*	*Ixodes ricinus*	Blackbird	*B. garinii*	2019
OP895972	*groEL*	*Ixodes ricinus*	Blackbird	*B. valaisiana*	2019
OP895973	*groEL*	*Ixodes ricinus*	Blackbird	*B. valaisiana*	2019
OP895974	*groEL*	*Ixodes ricinus*	Blackbird	*B. valaisiana*	2019
OP895975	*groEL*	*Ixodes ricinus*	Blackbird	*B. valaisiana*	2019
OP895976	*groEL*	*Ixodes ricinus*	Blackbird	*B. valaisiana*	2019
OP895977	*groEL*	*Ixodes ricinus*	Blackbird	*B. garinii*	2019
OP895978	*groEL*	*Ixodes ricinus*	Blackbird	*B. valaisiana*	2019
OP895979	*groEL*	*Ixodes ricinus*	Blackbird	*B. valaisiana*	2019
OP895980	*groEL*	*Ixodes ricinus*	Blackbird	*B. valaisiana*	2019
OP895981	*groEL*	*Ixodes ricinus*	Blackbird	*B. valaisiana*	2019
OP895982	*groEL*	*Ixodes ricinus*	Blackbird	*B. garinii*	2019
OP895983	*groEL*	*Ixodes ricinus*	Blackbird	*B. garinii*	2019

OP895984	*groEL*	*Ixodes ricinus*	Blackbird	*B. garinii*	2019
OP895985	*groEL*	*Ixodes ricinus*	Blackbird	*B. garinii*	2019
OP895986	*groEL*	*Ixodes ricinus*	Blackbird	*B. garinii*	2019
OP895987	*groEL*	*Ixodes ricinus*	Blackbird	*Borrelia sp.*	2019
OP895988	*groEL*	*Ixodes ricinus*	Blackbird	*Borrelia sp.*	2019
OP895992	*groEL*	*Ixodes ricinus*	Blackbird	*B. garinii*	2019
OP895993	*groEL*	*Ixodes ricinus*	Blackbird	*B. garinii*	2019
OP895994	*groEL*	*Ixodes ricinus*	Blackbird	*B. afzelii*	2019
OP895995	*groEL*	*Ixodes ricinus*	Blackbird	*B. afzelii*	2019
OP895996	*groEL*	*Ixodes ricinus*	Blackbird	*Borrelia sp.*	2019
OP895997	*groEL*	*Ixodes ricinus*	Blackbird	*B. garinii*	2019
OP895998	*groEL*	*Ixodes ricinus*	Blackbird	*B. garinii*	2019
OP896008	*groEL*	*Ixodes ricinus*	Blackbird	*B. garinii*	2019
OP896009	*groEL*	*Ixodes ricinus*	Blackbird	*Borrelia sp.*	2019
OP895999	*groEL*	*Ixodes ricinus*	Blackbird	*B. garinii*	2019
OP896010	*groEL*	*Ixodes ricinus*	Blackbird	*B. valaisiana*	2019
OP896011	*groEL*	*Ixodes ricinus*	Blackbird	*B. garinii*	2019
OP896000	*groEL*	*Ixodes ricinus*	Blackbird	*B. garinii*	2019
OP896001	*groEL*	*Ixodes ricinus*	Blackbird	*B. garinii*	2019
OP896012	*groEL*	*Ixodes ricinus*	Blackbird	*B. garinii*	2019
OP896002	*groEL*	*Ixodes ricinus*	Blackbird	*B. valaisiana*	2019
OP896013	*groEL*	*Ixodes ricinus*	Blackbird	*B. garinii*	2019
OP896003	*groEL*	*Ixodes ricinus*	Blackbird	*B. garinii*	2019
OP896004	*groEL*	*Ixodes ricinus*	Blackbird	*B. garinii*	2019
OP896014	*groEL*	*Ixodes ricinus*	Blackbird	*B. valaisiana*	2019
OP896005	*groEL*	*Ixodes ricinus*	Blackbird	*B. garinii*	2019
OP896006	*groEL*	*Ixodes ricinus*	Blackbird	*B. garinii*	2019
OP896015	*groEL*	*Ixodes ricinus*	Redwing	*B. garinii*	2019
OP896007	*groEL*	*Ixodes acuminatus*	Redwing	*Borrelia sp.*	2019
OP896016	*groEL*	*Ixodes ricinus*	Song thrush	*B. valaisiana*	2020
OP896017	*groEL*	*Ixodes ricinus*	Chaffinch	*B. afzelii*	2020

OP896018	*groEL*	*Ixodes ricinus*	Chaffinch	*B. afzelii*	2020
OP896019	*groEL*	*Ixodes ricinus*	Blackbird	*B. garinii*	2020
OP896020	*groEL*	*Ixodes ricinus*	Blackbird	*B. garinii*	2020
OP896021	*groEL*	*Ixodes ricinus*	Blackbird	*B. garinii*	2020
OP896022	*groEL*	*Ixodes ricinus*	Blackbird	*B. garinii*	2020
OP896023	*groEL*	*Ixodes ricinus*	Blackbird	*B. garinii*	2020
OP896024	*groEL*	*Ixodes ricinus*	Blackbird	*B. burgdorferi*	2020
OP896025	*groEL*	*Ixodes ricinus*	Blackbird	*B. garinii*	2020
OP896026	*groEL*	*Ixodes ricinus*	Redwing	*B. garinii*	2020
OP896027	*groEL*	*Ixodes ricinus*	Redwing	*B. garinii*	2020
OP896028	*groEL*	*Ixodes ricinus*	Redwing	*B. garinii*	2020
OP896029	*groEL*	*Ixodes ricinus*	Blackbird	*B. valaisiana*	2020
OP896049	*groEL*	*Ixodes ricinus*	Blackbird	*Anaplasma phagocytophilum* (ET 2)	2019
OP896050	*groEL*	*Ixodes ricinus*	Blackbird	*Anaplasma phagocytophilum* (ET 1)	2019
OP896052	*groEL*	*Ixodes ricinus*	Brambling	*Anaplasma phagocytophilum* (ET 2)	2019
OP896051	*groEL*	*Ixodes ricinus*	Blackbird	*Anaplasma phagocytophilum* (ET 1)	2020
OP890295	*16S rRNA*	*Ixodes ricinus*	Blackbird	Candidatus *Neoehrlichia mikurensis*	2020
OP890296	*16S rRNA*	*Ixodes ricinus*	European robin	Candidatus *Neoehrlichia mikurensis*	2020
OP890297	*16S rRNA*	*Ixodes ricinus*	Blackbird	*E. muris*	2019
OP896034	*gltA*	*Ixodes ricinus*	Blackbird	*R. helvetica*	2019
OP896035	*gltA*	*Ixodes ricinus*	Chaffinch	*R. helvetica*	2019
OP896036	*gltA*	*Ixodes ricinus*	Brambling	*R. helvetica*	2019
OP896037	*gltA*	*Ixodes ricinus*	European robin	*R. helvetica*	2019
OP896038	*gltA*	*Ixodes ricinus*	European robin	*R. helvetica*	2019
OP896039	*gltA*	*Ixodes ricinus*	Blackbird	*R. helvetica*	2019
OP896040	*gltA*	*Ixodes ricinus*	Blackbird	*R. helvetica*	2019
OP896041	*gltA*	*Ixodes ricinus*	Blackbird	*R. helvetica*	2019
OP896042	*gltA*	*Ixodes ricinus*	Blackbird	*R. helvetica*	2019
OP896043	*gltA*	*Ixodes ricinus*	Blackbird	*R. helvetica*	2019
OP896044	*gltA*	*Ixodes ricinus*	Blackbird	*R. helvetica*	2019
OP896045	*gltA*	*Ixodes ricinus*	Blackbird	*R. helvetica*	2019

OP896046	*gltA*	*Ixodes ricinus*	Blackbird	*R. helvetica*	2019
OP896047	*gltA*	*Ixodes ricinus*	Redwing	*R. helvetica*	2019
OP896048	*gltA*	*Ixodes ricinus*	Brambling	*R. helvetica*	2019

**Accession number**	**Gene**	**Tick Species ID**	**Bird host**	**Year**
OR499166	*16S rRNA*	*Ixodes ricinus*	Song trush	2019
OR499167	*16S rRNA*	*Ixodes ricinus*	European robin	2020
OR499168	*16S rRNA*	*Ixodes ricinus*	European robin	2020
OR499169	*16S rRNA*	*Ixodes ricinus*	Blackbird	2019
OR499170	*16S rRNA*	*Ixodes ricinus*	Blackbird	2019
OR499171	*16S rRNA*	*Ixodes ricinus*	Blackbird	2020
OR499172	*16S rRNA*	*Ixodes ricinus*	Blackbird	2020
OR499173	*16S rRNA*	*Ixodes ricinus*	Brambling	2019
OR499174	*16S rRNA*	*Ixodes ricinus*	Brambling	2019
OR499175	*16S rRNA*	*Ixodes ricinus*	Blackbird	2019
OR499176	*16S rRNA*	*Ixodes ricinus*	Blackbird	2019
OR499177	*16S rRNA*	*Ixodes ricinus*	Brambling	2019
OR499178	*16S rRNA*	*Ixodes frontalis*	European robin	2019
OR499179	*16S rRNA*	*Ixodes ricinus*	Blackbird	2019
OR499180	*16S rRNA*	*Ixodes ricinus*	Redwing	2019
