# Supplementary material for: Survey of Tick-Borne Zoonotic Agents in Ixodes Ticks Carried by Wild Passerines during Postbreeding Migration through Italy
Source: Transbound Emerg Dis. 2023 Nov 14;2023:1399089. doi: 10.1155/2023/1399089 (PMC12016753; doi:10.1155/2023/1399089)
Supplement: Supplementary 3 — Ringed bird species during season 2019, 2020, and total count. Details are provided about the partial and total number of bird species and the quantity of individuals/species. Tick infested bird species were orange-labelled. [file 1399089.f3.pdf]

Species		Ringed birds		
Common name	Scientific name	**2019**	**2020**	**Total**
Black grouse	*Tetrao tetrix*	2	0	2
Alpine rock partridge	*Alectoris Graeca*	2	1	3
Sparrowhawk	*Accipiter nisus*	4	0	4
Jack Snipe	*Lymnocryptes minimus*	1	0	1
Woodcock	*Scolopax rusticola*	3	3	6
Pygmy Owl	*Glaucidium passerinum*	1	0	1
Long-eared Owl	*Asio otus*	0	1	1
Tengmalm's Owl	*Aegolius funereus*	1	3	4
Grey-faced Woodpecker	*Picus canus*	2	0	2
Black woodpecker	*Dryocopus martius*	1	1	2
Great Spotted Woodpecker	*Dendrocopos major*	7	0	7
Tree Pipit	*Anthus trivialis*	0	1	1
Meadow Pipit	*Anthus pratensis*	7	1	8
Water Pipit	*Anthus spinoletta*	42	7	49
Winter Wren	*Troglodytes troglodytes*	4	4	8
Hedge Accentor	*Prunella modularis*	19	32	51
European Robin	*Erithacus rubecula*	776	197	973
Black Redstart	*Phoenicurus ochruros*	68	62	130
Common Redstart	*Phoenicurus phoenicurus*	10	2	12
Northern Whetear	*Oenanthe oenanthe*	1	0	1
Eurasian Blackbird	*Turdus merula*	67	20	87
Fieldfare	*Turdus pilaris*	1	0	1
Song Thrush	*Turdus philomelos*	65	12	77
Redwing	*Turdus iliacus*	14	1	15
Mistle Thrush	*Turdus viscivorus*	27	3	30
Blackcap	*Sylvia atricapilla*	2	1	3
Common Chiffchaff	*Phylloscopus collybita*	11	5	16
Goldcrest	*Regulus regulus*	403	119	522
Firecrest	*Regulus ignicapillus*	3	1	4
Blue Tit	*Cyanistes caeruleus*	12	0	12
Great Tit	*Parus major*	5	1	6
Coal Tit	*Periparus ater*	884	13	897
Willow tit	*Poecile montanus*	0	1	1
Marsh Tit	*Poecile palustris*	2	1	3
Short-toed Tree-Creeper	*Certhia brachydactyla*	1	0	1
Eurasian Jay	*Garrulus glandarius*	1	0	1
Spotted Nutcracker	*Nucifraga caryocatactes*	4	0	4
Chaffinch	*Fringilla coelebs*	104	37	141
Brambling	*Fringilla montifringilla*	90	3	93
European Goldfinch	*Carduelis carduelis*	4	0	4
Eurasian Siskin	*Carduelis spinus*	63	135	198
Red Crossbill	*Loxia curvirostra*	1	2	3
Eurasian Bullfinch	*Pyrrhula pyrrhula*	4	0	4
Hawfinch	*Coccothraustes coccothraustes*	1	4	5
Rock Bunting	*Emberiza cia*	14	2	16
Reed Bunting	*Emberiza schoeniclus*	0	1	1
**Total ringed birds**		2.734	677	3411
**Total ringed species**		42	32	46
