# Supplementary figures and images for: Survey of Tick-Borne Zoonotic Agents in Ixodes Ticks Carried by Wild Passerines during Postbreeding Migration through Italy
Source: Transbound Emerg Dis. 2023 Nov 14;2023:1399089. doi: 10.1155/2023/1399089 (PMC12016753; doi:10.1155/2023/1399089)

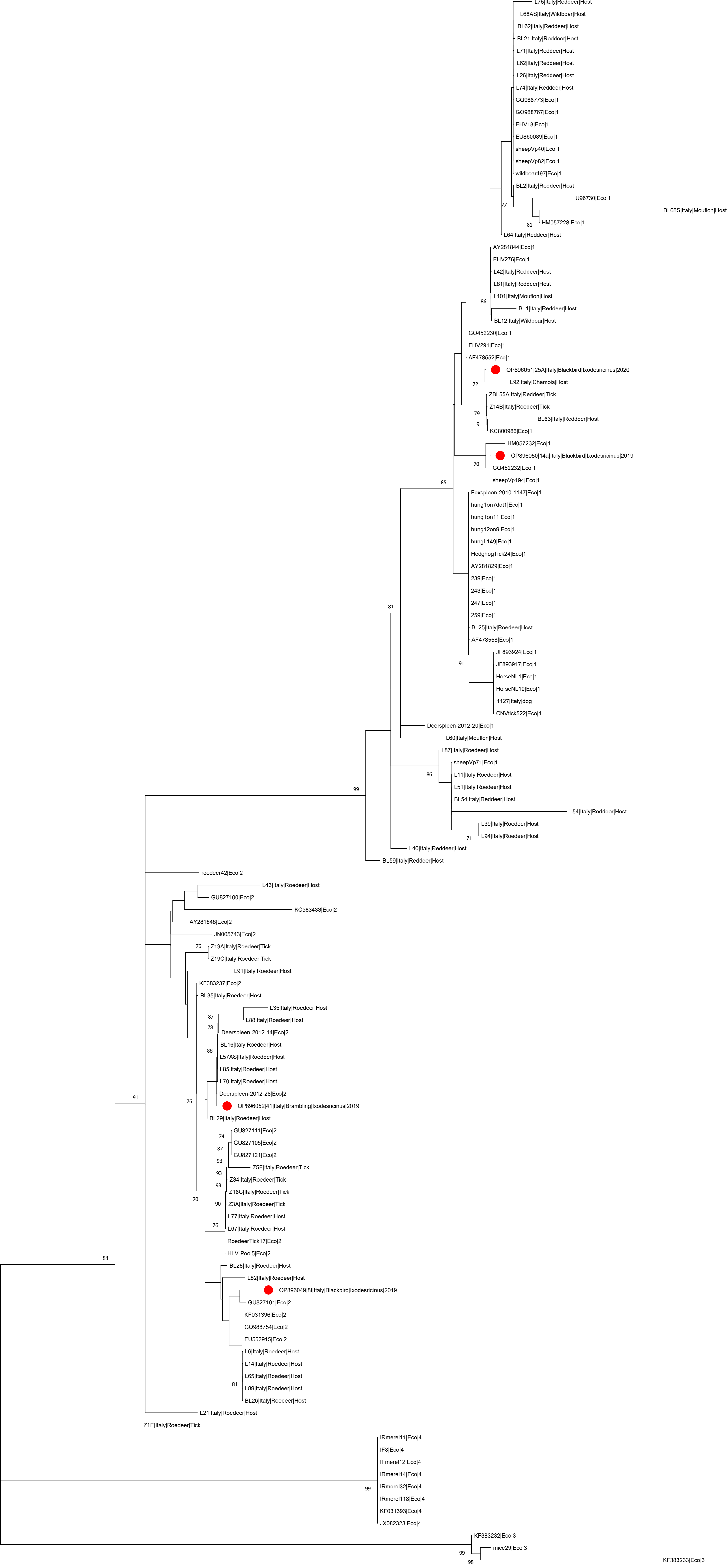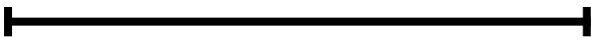

0.01

Supplement: Supplementary 4 — Anaplasma phagocytophilum phylogenetic tree. The evolutionary tree was inferred using the neighbor-joining method on a dataset of partial groEL sequences. The confidence probability was estimated using the bootstrap test. Only values higher than 70% are reported. The strains identified in the present study are highlighted by red circles. [file 1399089.f4.pdf]

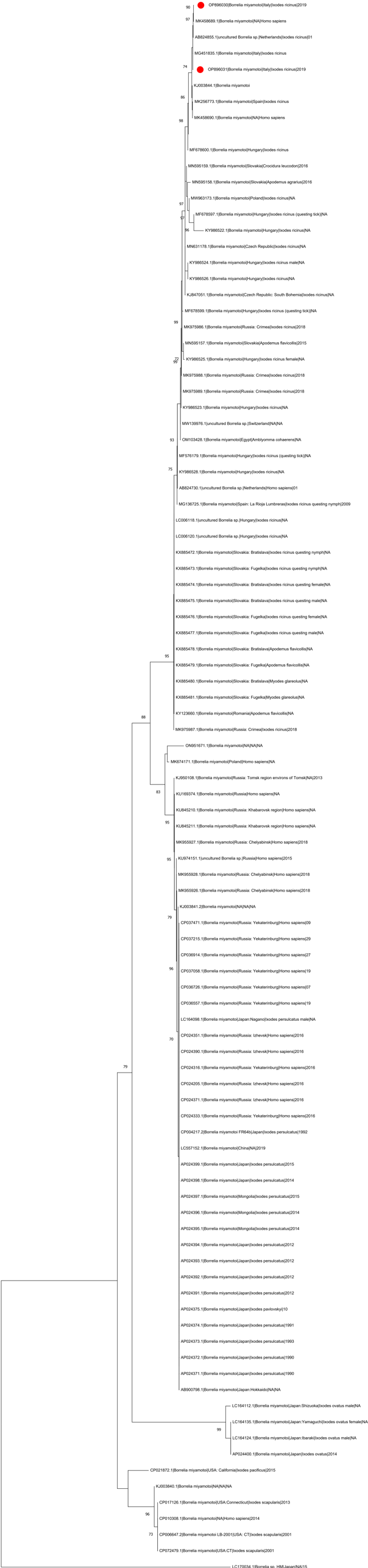

0.01

Supplement: Supplementary 5 — Borrelia miyamotoi phylogenetic tree. The evolutionary tree was inferred using the neighbor-joining method on a dataset of partial groEL sequences. The confidence probability was estimated using the bootstrap test. Only values higher than 70% are reported. The strains identified in the present study are magnified in the right insert and highlighted by red circles. [file 1399089.f5.pdf]
